# 

**Published:** 1835-04-01

**Authors:** 


### NOTICES.

*Our Readers will observe, that, in order to make room for a considerable quantity of interesting foreign matter, we have given twenty-four pages extra in our present Number.*

*It is with great pleasure that we announce to our readers, that* G. T. BURNETT, Esq., *Professor of Botany at King's College, was elected Professor of Botany to the Society of Apothecaries, on the* 17*th of March.*

*We regret that we cannot insert the Review of* ANDRAL: *it shall be left for the author at our publisher's.*

*The conclusion of* DR. STROUD's *Paper is unavoidably postponed, for want of room.*

*We have received the following Works:*

TWINING *on the Diseases of Bengal.*
COWAN's *Translation of* LOUIS *on Phthisis.*
ROYLE's *Himalayan Plants.* Part V.
WEATHERHEAD *on Rickets.* Second Edition.
QUAIN's *Anatomical Plates.* Fasc. XXII. XXIII. *and* XXIV.
HOUSTON's *Catalogue of the Museum of the Royal College of Surgeons in Ireland.*
GREEN *on Diseases of the Skin.*
*Cyclopædia of Medicine.* Part XXV.
HODGKIN *on the Means of Preserving Health.*
MURRAY's *Manual of Chemical Experiments.* Fourth Edition.
GRANT's *Comparative Anatomy.* Part I.
NEILL's *Ophthalmic Report.*
DESBERGER's *Marriage Almanack.*

*We shall review several of them in our next.*

### BRITISH AND FOREIGN MEDICAL REVIEW.

*On the 1st January, 1836, will be published, by Messrs. Sherwood and Co.*
*Price Six Shillings,*

THE FIRST NUMBER

OF

### THE BRITISH AND FOREIGN
### Medical Review;

OR,

### QUARTERLY JOURNAL

OF

### PRACTICAL MEDICINE AND SURGERY.

EDITED BY

JOHN FORBES, M.D. F.R.S.

AND

JOHN CONOLLY, M.D.

***Editors of the Cyclopædia of Practical Medicine.***

### PROSPECTUS.

Four years of active Editorial communication with numerous correspondents holding a very high rank in the profession, and practising it in various parts of the Empire, have led to the conviction that the Cyclopædia of Practical Medicine, recently brought to a conclusion, will be most appropriately and usefully followed by a Quarterly Review and Journal of the Medical Sciences; serving, among other purposes, the valuable one of appending to that work the continual additions making to Practical Medicine, and carrying still more widely into effect the diffusion of information throughout the profession than was consistent with the defined plan of that publication, on the merits of which the public have pronounced so favorable a decision.

It is proposed to effect these objects by the publication of The British and Foreign Medical Review, which will be conducted by two of the Editors of the Cyclopædia. In the department of Practical Medicine it will be enriched by the contributions of several of the writers whose assistance was given to that work, and by other physicians of like talent and experience; whilst in that of Practical Surgery, and in the various other branches of medical science, the co-operation of many labourers has been obtained whose names are equally well known to the public, and, if announced, would give the most satisfactory assurance of the honorable principles by which their critical duties will be guided.

There seems the fullest reason to believe, notwithstanding the great and acknowledged ability displayed in the medical periodical press, that, in the actual state of medical literature, a work of higher critical aims than are professed by the conductors of the principal journals in this country, is really desired by the members of the medical profession. It is, therefore, intended that the notices of books contained in the British and Foreign Review, shall not be confined to a mere analysis of their contents, or the placing before the unstudious reader considerable portions of them in the shape of extracts connected by a slender and negligent commentary. Whilst one constant object of the Editors will be to convey as much valuable information as possible, they will feel it incumbent upon them to cause that information to be accompanied with such free and candid critical discussion as may be furnished by writers possessing a competent knowledge of the previous Literature of the subject of each article, or as the work reviewed may demand. This course, they apprehend, will be equally satisfactory to the reader, and to the author reviewed.

The execution of these Critical and Analytical Reviews will, it is hoped, evince that they are the productions of men of experience and of efficient acquirements, independent of the influences too evident in the hasty productions of juvenile and inconsiderate writers. It has often been a most just source of complaint on the part of authors that this important task has been confided to persons little qualified to undertake it. The liberal learning and the arduous efforts of the cultivators of a difficult profession can never be encouraged, cannot even be appreciated, by inexperienced reviewers, however able; and the thoughtful productions of half a life of observation may be as easily misrepresented in consequence of the limited knowledge of the critic, as of his want of candour and an exercised judgment. Writers of known and unquestionable respectability will, it is presumed, be less anxious to display critical severity than to examine with care the real pretensions of the books which fall under their observation; and, not being unacquainted with the claims of previous authors, will be best enabled to state with fairness and clearness the merits of the works they undertake to criticise.

But as with the present facilities of publication, the productions of the press are exceedingly numerous, and many of them, although not destitute of merit, are yet ephemeral in their character, and so limited in their intention as rather to require brief mention than elaborate review, a considerable portion of the British and Foreign Medical Review will be devoted to such Bibliographical Notices as may best comprehend the most recent facts and speculations recorded in the essays, pamphlets, theses, and minor publications of this and other countries. It is unnecessary to dwell on the obvious advantages of this plan to readers whose time is much occupied by professional duties, and to purchasers of books who feel it necessary only to *select* from the number daily advertised.

A distinguishing feature of the Review will be the extent and fullness of its foreign department, in which it is intended to present an exposition of the actual and progressive medical literature of all the countries of Europe in which such a literature can be properly said to exist. Since the discontinuance, in 1823, of the Quarterly Journal of Foreign Medicine and Surgery, a work of great, although of unequal merit, and which was very valuable to the student, this department has never been sufficiently regarded; and of the state of medical knowledge abroad, with the single exception of France, the English reader is far from being adequately informed. For carrying this part of the plan into effect, very extensive arrangements have been made with accomplished medical scholars at home, and with physicians and surgeons in most other parts of the world in which medicine and surgery are cultivated. Particular attention will of course be given to the state and advancement of medical science in countries especially distinguished by the zeal, activity, and proficiency of its professors, as France, Germany and Italy; but the literature of the other countries of Europe, in which medicine either is, or is considered to be, less advanced, as in Russia, Poland, Sweden, Denmark, Spain and Portugal, will be systematically observed and reported upon. The overflowing stores of the more favoured countries may usefully be contrasted with the comparative poverty and destitution of others; science may even be advanced by a contemplation of the causes of such striking diversities; and general views may be enlarged and strengthened by an attention to the consequences of different stages of the progress towards rational medicine, on the health and comfort of mankind.

The advancing state of medical science in America, and the zeal of British observers and investigators in the different Colonies, will receive all the consideration which the important results that are to be expected from them may be found to demand. The improving condition of Oriental Medicine is opening a new field of comparison and inquiry; and it will be found not uninteresting to survey the progress of the sciences connected with the healing art in countries towards which every kind of knowledge for which those regions were once before celebrated, is now proceding from the West, after a long æra during which all the splendours of science have in those regions been obscured. Egypt and Turkey may be mentioned as already beginning to contribute to the stock of medical knowledge.

In the proceedings of the well organized Medical Societies of the Continent, valuable information and interesting intelligence is often to be found, which will deserve and find a place in a small department of the British and Foreign Medical Review, set apart for MISCELLANEOUS MEDICAL INTELLIGENCE. The same department will comprehend such official documents as may be important or useful to the profession; new legal enactments affecting medical men;—new regulations of any of the Medical Corporations; occasional reports of the proceedings of Societies at home;—scientific notices bearing on medicine;—&c. &c. &c.

It is not intended that the pages of the Review shall contain original communications, except in the form of Critical essays; or any reports of cases, or extracts from books or journals published in Great Britain, except in the shape of Analytical or Bibliographical notices.

The exceeding value given to works of modern date by the recent progress and great advancement of anatomy, physiology and pathology, has had a tendency to produce an impatience of reference to the folios of the earlier authorities, or even to their less voluminous writings, in which the valuable facts too often lie concealed in a multitude of words perplexing and unsatisfactory to the reader. Yet the originality of the older authors, and their excellence as observers, and the practical truths to which by observation they attained, deserve better of modern times than to be lost in an ungrateful obscurity. One legitimate object, therefore, of Critical composition, is to revert to the ancient learning of the profession, and to bring it before the modern reader without the inconveniences which have been alluded to. With this view, and with the desire, when it can be satisfactorily done, of tracing medical truths to their actual origin, or of illustrating portions of rare or almost forgotten medical literature, it is extremely desirable that room should occasionally be found for RETROSPECTIVE REVIEWS of works which once enjoyed a just celebrity, and are still worthy of being remembered. The space devoted to such papers will necessarily be regulated by various considerations which it is unnecesary here to specify.

The size and form of the British and Foreign Medical Review will be that which has been found most suitable to Quarterly publications, whether of medical or general literature; and sufficient to afford space for full reviews of several publications, exclusive of Bibliographical notices and Intelligence, in each number. Two numbers will form a volume.

Of the general plan of the proposed Review it is unnecessary to say more in a Prospectus; but a few words may be added concerning the spirit in which the Editors propose to conduct it. Both of them are privileged at least to say that they undertake their duty unfettered by any considerations which can affect their independence; and, they trust, by any prejudices which can affect their impartiality. The articles to which they give admission in the Review must necessarily be the work of writers variously situated, and of various habits of feeling and of thought; but each article will be carefully revised by the Editors before publication, and they will consider themselves solely responsible for the manner, although they cannot always be so for the matter of each contribution. Their views of the duties they are commencing have been well considered, and they may add, perhaps, corrected by a considerable experience, arising out of their engagements with the Cyclopædia, of the actual state of literary efficiency or defect united with various degrees of medical acquirements.

A reviewer, they conceive, should be more anxious to do justice to the merits than to disclose the faults of the writers whose works he professes to review. He should entertain a just respect for industry, and a due consideration of the numerous sources of error which pour their deceitful waters across the difficult path of truth. As much as possible, he should be free from strong predilections or prejudices relative to theories or to men, and of a temper not given to harbour ungenerous and blinding enmities. The first object of his desire should be the improvement of the knowledge and resources, and the maintenance of the character of his profession: nor can any thing well be considered to justify the exercise of his censorial severity, except unjustifiable, because avoidable ignorance, literary or scientific, fraudulent attempts upon older writers, or assertions which are not strictly veracious, or the low and knavish arts of quackery.

The critic constitutes himself a judge; and the first attribute of a judge is justice, which mercy should ever temper. He should doubtless check the presumptuous, but he should encourage the diffident; and he may applaud the bold, whilst he chastises the unprincipled. Exposed by his duties to the remonstrances of vanity, and the persuasions of those whom feelings of delicacy do not restrain, he may disregard both so long as he is conscious of no partiality or favour, save for what is honest and what is just; so long as his praise is willingly accorded to various degrees of merit, and his census reserved for presumptuous ignorance, and for all the disguises of imposture. And of all the parts of his duty, he should ever remember, that it is the highest and the most useful to discover or introduce, and to do willing honor to, whatever is excellent, whatever is ingenious, or whatever promises to be beneficial to the profession and to the public.

---

Communications and Books for review to bead dressed to the Editors, care of Messrs. Sherwood and Co. Paternoster Row, or to Dr. Forbes, Chichester, or Dr. Conolly, Warwick.

*Mason, Printer.*

